# Large Electrostrain and High Optical Transmittance via Oxygen Octahedron Rotation Disordering in KNN Ceramics

**DOI:** 10.1002/advs.77952

**Published:** 2026-10-11

**Authors:** Xia Luo, Jian Wang, Yu Tan, Jiyang Xie, Thamil Selvi Velayutham, Wanbiao Hu

**Affiliations:** ^1^ Yunnan Key Laboratory of Electromagnetic Materials and Devices National Center for International Research on Photoelectric and Energy Materials School of Materials and Energy Yunnan University Kunming People's Republic of China; ^2^ Southwest United Graduate School Kunming People's Republic of China; ^3^ School of Engineering Yunnan University Kunming People's Republic of China; ^4^ Low Dimensional Materials Research Centre (LDMRC) Department of Physics Faculty of Science Universiti Malaya Kuala Lumpur Malaysia

**Keywords:** asymmetric electrostrain, atomic‐scale characterization, lattice disordering, transparent ceramics

## Abstract

Lead‐free K_0.5_Na_0.5_NbO_3_ (KNN)‐based ceramics are promising candidates for multifunctional electronic materials. Nevertheless, the synergistic optimization of electrostrain and optical transmittance in KNN polycrystalline ceramics remains a critical challenge, owing to the structural trade‐off between large strain and high transparency. Here, we employ chemical disorder engineering specifically through BaTiO_3_ (BT) substitution to manipulate the BO_6_ oxygen octahedron rotation disorder. BT incorporated into KNN gradually modulates the BO_6_ rotation mode from local ordering to a random distribution, which refines the size of cubic‐phase‐enriched polar nanoregions, facilitating high transparency in visible‐light range. The disordered BO_6_ rotation essentially lowers the thermodynamic switching barriers for polarization and strengthens the defect‐dipole pinning effect, thereby enhance the electrostrain synergistically. The optimal composition, i.e., KNN‐0.03BT exhibits a large electrostrain of 1.2% and an optical transmittance of 60% in visible‐light range. By overcoming the long‐standing bottleneck in traditional polycrystalline ceramics, this work elucidates the core mechanism of atomic‐scale disordering‐enhanced polarization response and reduced optical loss, thereby advancing multifunctional material applications such as transparent sensing and flexible electronics.

## Graphical abstract

Chemical disorder engineering reconfigures BO_6_ oxygen‐octahedron rotations in lead‐free K_0.5_Na_0.5_NbO_3_ ceramics from local ordering into a random distribution, dissolving the longstanding trade‐off between optical transparency and electromechanical strain. The optimized KNN‐0.03BT composition delivers ∼ 60% visible‐light transmittance together with a giant electrostrain of 1.2%, opening a new route to transparent, multifunctional piezoelectrics.

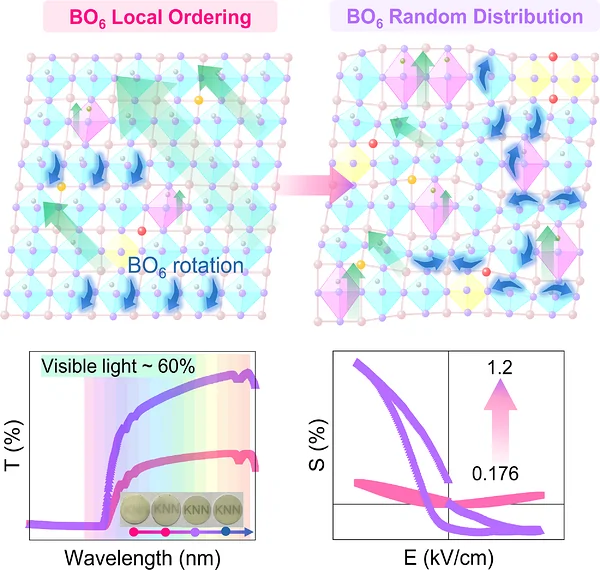

## 1 Introduction

Hybrid ultrasound–optical devices, e.g., photoacoustic imaging transducers and acoustic haptic actuators, call for transparent piezoelectrics [1, 2, 3]. However, it has long been a challenge to achieve high piezoelectricity and transparency simultaneously because the high‐performance piezoelectrics usually contain high‐density light‐scattering domain walls or grain boundaries [4, 5, 6]. Driven by increasingly stringent environmental regulations restricting lead‐based piezoelectric materials (e.g., PMN‐PT) [1, 4], lead‐free K_0.5_Na_0.5_NbO_3_ (KNN)‐based ceramics have emerged as a promising candidate alternative due to their high Curie temperature, favorable piezoelectric response, and environmental benignity [7, 8, 9]. However, achieving synergistic performance in KNN ceramics presents a fundamental trade‐off: high optical transparency demands an ordered microstructure with minimal light scattering, whereas a large strain response depends on symmetry breaking and domain engineering [6, 10]. These conflicting structural requirements render the simultaneous realization of both properties elusive, presenting a critical bottleneck to the practical deployment of KNN‐based opto‐electrostrain integrated devices [2, 6].

Currently, transparent KNN ceramics are mainly prepared via densification sintering, multiscale reconfiguration (e.g., grain and domain, grain boundary engineering), and single crystal growth to reduce optical anisotropy and light scattering [4, 6, 10, 11]. However, their electrostrain properties tend to degrade significantly after transparency optimization [12, 13, 14]. On the other hand, strategies such as the alignment control of defect dipoles [15, 16], domain structure engineering [17], internal bias field induction [18], and chemical piezoelectric effect [19] have offered avenues to achieve giant electrostrain. Nevertheless, these ceramics are optically opaque [20, 21, 22]. Strategies that can simultaneously achieve high optical transparency and a robust electrostrain response remain to be explored.

Octahedral rotation, as a fundamental structural degree of freedom in perovskites, governs lattice symmetry, polarization dynamics, and energy landscape, thereby playing a crucial role in determining electrostrain behavior and optical responses [23, 24, 25]. In particular, modulation of rotational configurations from local ordering to disordered states can disrupt structural coherence, facilitate polarization switching, and reduce optical anisotropy, offering a potential pathway toward simultaneously enhanced electrostrain and transparency [23, 26]. However, achieving such synergistic optimization remains challenging, and the underlying coupling mechanism between asymmetric electrostrain and optical transparency is still poorly understood [22, 26, 27]. In contrast to conventional approaches based on compositional design or domain engineering, regulation of octahedral rotation provides an intrinsic and structure‐centric strategy [28, 29]; yet its role in mediating multi‐scale structural disorder and its impact on the correlation between electrostrain and optical properties have only recently begun to be explored, leaving significant scientific questions to be addressed [15].

In this study, KNN ceramics doped with 2 at.% Dysprosium (Dy) are employed as the model system. Multiple types of atomic‐scale structural disorders are introduced via BaTiO_3_, with the disorder degrees regulated accordingly. Constructing A‐site compositional disorder enhanced B‐site displacement vectors, oxygen octahedral rotations, and lattice parameter variations. This refined the PNRs while maximizing polarization intensity, ultimately inducing asymmetric electrostrain. Concurrently, reduced lattice anisotropy enhances the optical transmittance of the ceramics. This study aims to systematically elucidate the atomic‐scale mechanism underlying the synergistic optimization of these two properties and realize the simultaneous enhancement of asymmetric electrostrain and optical transmittance in KNN ceramics.

## 2 Results and Discussion

### 2.1 Crystal Structure and Defect States

(1‐*x*)(K_0.5_Na_0.5_)_0.98_Dy_0.02_NbO_3_‐*x*BaTiO_3_ (*x* = 0.005, 0.01, 0.03, 0.05, abbreviated as KNN‐*x*BT) ceramics were fabricated via a solid‐state reaction method. The (200) diffraction peak near 45° was locally magnified and deconvoluted, achieving well‐fitted profiles, as presented in Figure 1a. The distinctly asymmetric contour of the (200) peak verifies the mixed‐phase nature of this system [8, 30]. Figure 1b displays the Rietveld‐refined XRD patterns of the KNN‐0.03BT sample recorded over 2θ = 10°‐90°, with structural refinement conducted using the *Amm2* and *P4mm* space groups. Figure 1c summarizes the phase proportions of all as‐prepared compositions. Rietveld fitting results confirm the coexistence of tetragonal and orthorhombic phases with an equal molar ratio of 50% each, demonstrating that K^+^, Na^+^, Nb^5+^, Ba^2+^, Ti^4+^, and Dy^3+^ ions have fully diffused into the host KNN lattice. The obtained perovskite structure for KNN‐0.03BT maintains favorable thermal stability from 20°C to 450°C (Figure S1b). Raman spectra (Figure S2) show no impurity peaks introduced by BT doping. Meanwhile, both the full width at half maximum (FWHM) and peak positions of the *v_1_* and *v_2_* modes (Figure 1d,e), which correspond to octahedral breathing vibrations, exhibit no remarkable variations. X‐ray photoelectron spectroscopy (XPS) was employed to characterize the oxidation states and ionic vacancy behavior (Figure 1f,h). No noticeable intensity changes are observed for the K 2*p*_3/2_ and K 2*p*_1/2_ characteristic peaks in Figure 1f, indicating that BT doping exerts a negligible effect on potassium vacancy content. Previous literature has proven that elevated K 2*p* peak intensities are strongly correlated with increased potassium vacancies [31]. In the O 1s spectra (Figure 1g), the main peak centered at 529.5 eV corresponds to lattice oxygen, whereas the higher‐binding‐energy shoulder at 531.5 eV is assigned to adsorbed oxygen associated with oxygen vacancies. There are no obvious differences in these two oxygen‐related peaks between the KNN‐0.005BT and KNN‐0.03BT samples. In addition, no detectable valence state evolution of Nb^5+^ is observed across the doping series (Figure 1h). Electron paramagnetic resonance (EPR) spectra collected under different attenuations (Figure 1i) remain EPR‐silent, further confirming the absence of unpaired electrons in the KNN‐0.03BT ceramic at room temperature. Combined XPS and EPR analyses confirm that moderate BT doping exerts minimal effect on potassium and oxygen vacancy concentrations, stabilizes the Nb^5+^ valence state, and suppresses the formation of additional defects and unpaired electrons in KNN‐based ceramics. As illustrated in Figure S3, KNN‐xBT ceramics possess fine grains with an average size of ∼ 190 nm and develop a disordered perovskite structure induced by multiphase coexistence. Such a structural feature provides a crucial foundation for the synergistic regulation of optical transparency and asymmetric electrostrain. Overall, within the investigated doping range, the introduction of BaTiO_3_ does not alter the intrinsic bulk crystal structure of the KNN 2 at.% Dy matrix.

**FIGURE 1:**
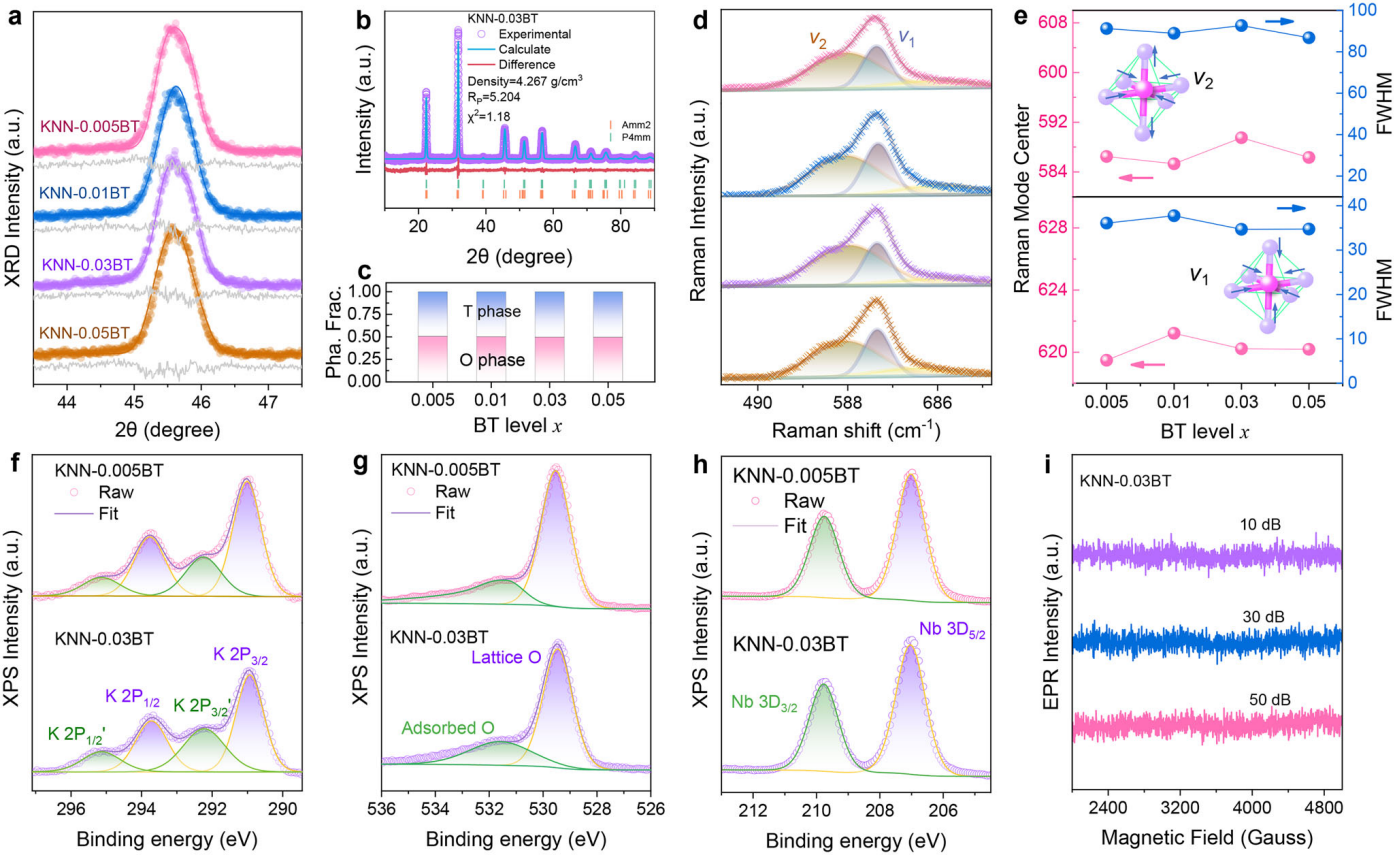
Structural and chemical state characterizations of KNN‐*x*BT based ceramics. (a) (200) diffraction peaks. (b) XRD Rietveld refinement. (c) Phase fraction (Pha. Frac.) derived from XRD refinement. (d) Raman spectra with deconvoluted vibrational modes and (e) parameters for *v_1_*, *v_2_* modes. (f‐h) XPS spectra for K 2*p*, O 1*s*, and Nb 3*d*, respectively. (i) EPR spectra at different attenuations (dB).

### 2.2 Polarization Configurations and Octahedral Rotation Disordering

The atomic‐scale structures of KNN‐0.005BT and KNN‐0.03BT ceramics were investigated using High Angle Annular Dark Field‐Scanning Transmission Electron Microscopy (HAADF‐STEM) along the [001]_C_ and [110]_C_ pseudocubic zone axes, revealing substantial variations in structural disorder and polarization configuration with increasing BT content. The HAADF‐STEM image along the [001]_C_ zone axis of KNN‐0.03BT (Figure 2a) shows distinct intensity variations for *A‐*site cations. Here, lattice displacement is defined as the polar offset of the B‐site cation from the geometric center of its four nearest‐neighboring A‐site cations (inset of Figure 2a). The corresponding *A‐*site line‐scan profile (Figure 2b) confirms the presence of *A‐*site vacancies (highlighted by dash circle) and heavy heteroatoms. Figure 2c compares the quantitative statistical histograms of normalized *A‐*site atomic intensities for KNN‐0.005BT and KNN‐0.03BT. In HAADF‐STEM column intensity analysis, a symmetric Gaussian distribution indicates a chemically ordered A‐sublattice with uniform atomic occupancy, whereas a skewed distribution signifies prominent local compositional fluctuations [32]. The evolution from a normal to an asymmetric distribution in KNN‐0.03BT confirms enhanced A‐site chemical disorder. Atomic‐scale elemental mapping further validates the random distribution of K/Na/Dy/Ba on A‐sites and Nb/Ti on B‐sites (Figure S4), which directly modulates the local chemical environment of the lattice [15, 33, 34]. To quantitatively characterize the local structural inhomogeneity, the distribution of unit cell parameters is statistically analyzed. Figure 2d compares the scatter plots of lateral *a* and longitudinal *c* unit cell parameters extracted from Figure S5a,c. It is evident from the plot that the KNN‐0.03BT exhibits broader dispersion in the lattice parameters, indicating the stronger structural disorder in this specimen. The polarization vectors of *B‐*site cations are determined by their displacements from the geometric centroid of four adjacent *A‐*site cations (inset in Figure 2a). The polarization vector distribution maps of KNN‐0.005BT and KNN‐0.0BT are present in Figure S5b,d. In KNN‐0.005BT, polarization vectors preferentially align along the [110]_C_ direction, while adopting multidirectional orientations in KNN‐0.03BT. The results indicate that although the crystal structure is macroscopically pseudocubic, local polar nanoregions (PNRs) emerge in the lattice, with their size declining as the BT concentration rises.

**FIGURE 2:**
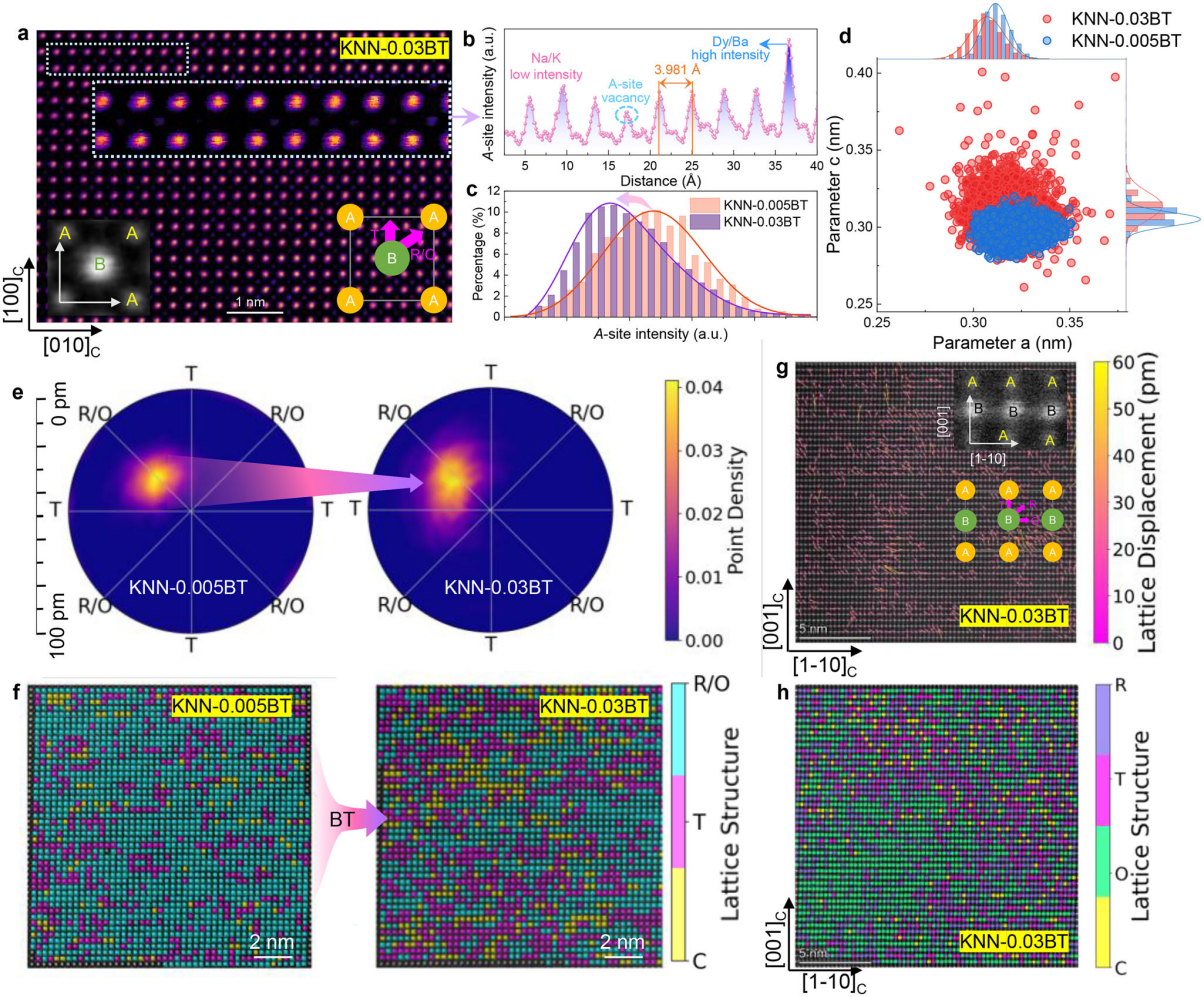
Atomic‐scale structure and local dipole polarization distributions for KNN‐0.005BT and KNN‐0.03BT obtained by HAADF‐STEM along the [001]_C_ and [110]_C_ zone axes. (a) HAADF‐STEM image along the [001]_C_, with insets showing the schematic definition for displacement fitting. (b) *A‐*site line intensity profile marked in (a) (light purple line). (c) Comparative statistical histograms of normalized *A‐*site atomic intensities. (d) Comparative statistical scatterplots of lattice parameters *a* and *c* along the [001]_C_. (e) Statistical distribution of the amplitude and angle of the polarization vector along the [001]_C_. (f) Spatial phase distributions mapped from [001]_C_ HAADF‐STEM images. (g) Atomic‐resolution HAADF‐STEM image and overlayed polarization vectors along the [110]_C_ direction, with insets showing schematic definition for fitting. (h) Phase distribution mapped from the [110]_C_ HAADF‐STEM image.

To more intuitively reflect the evolution of polarization vectors, the polarization vector density (Figure 2e) was extracted from Figure S5b,d. The radius of the polar plot in Figure 2e represents the magnitude of the displacement vector, and the angle indicates its direction. With rising BT doping, local PNRs shift from aggregation within the orthorhombic lattice to dispersion in the tetragonal phase, while the polarization magnitudes also display broader variations, demonstrating the enhanced disordering of B‐site polarization vectors. The phase map in Figure 2f reveals that KNN‐0.03BT possesses a significantly higher fraction of randomly distributed tetragonal (T) and pseudo‐cubic (C) local regions intermixed with the orthorhombic (R/O) matrix. This multi‐phase coexistence flattens the free‐energy landscape, effectively lowering the energy barrier for domain switching and polarization rotation [17]. Given that rhombohedral (R) and orthorhombic (O) phases overlap in projection on the [100]_C_ plane, polarization vectors in the [110]_C_ plane were further analyzed to unambiguously differentiate these two phases, which are shown in Figure 2g,h. Coexistence of R,O,T and C nanodomains flattens the energy barrier and gives rise to high structural heterogeneity or an isotropic polar energy landscape [35].

Figure 3a presents the atomic‐resolution iDPC‐STEM image of KNN‐0.03BT along the [001]_C_ direction, concurrently showing the projected bond lengths and unit cell characteristics, with the four projected BO6 octahedral plane clearly specified. Diverse BO_6_ octahedral rotation modes are visualized in Figure 3b. To quantitatively characterize local framework distortions, two descriptors are defined in Figure 3b,c: the octahedral rotation angle and the O_13_/O_24_ distortion parameter. As illustrated in Figure 3c, the O_13_/O_24_ parameter is determined from the four in‐plane oxygen sites (O_1_, O_2_, O_3_, O_4_). It measures the relative displacement difference between the vertical O_1_‐O_3_ and O_2_‐O_4_ atom pairs, directly reflecting oxygen‐sublattice distortion. Figure 3d,f presents the spatial distribution maps of BO_6_ octahedral rotation angles for the KNN‐0.005BT and KNN‐0.03BT samples, respectively. Although the rotation angles in KNN‐0.005BT display local fluctuations (Figure 3d), they retain strong spatial correlation. Within localized regions, both the direction and magnitude of the octahedral tilts remain continuous and coordinated, confirming a locally ordered rotation framework. In contrast, the rotation angle distribution in the KNN‐0.03BT sample (see Figure 3f) becomes significantly more fragmented and dispersed, with more frequent alternation between positive and negative rotations across different regions and a more irregular color variation. This indicates that the octahedral rotations no longer maintain clear local cooperativity but instead evolve toward a more random distribution. This provides direct real‐space evidence for the transition from local ordering to spatial disorder. Figure 3e shows the corresponding statistical distribution of octahedral rotation angles. The results indicate that the BO_6_ rotation angles are generally confined within a narrow range, exhibiting slight deviations around 0°, suggesting that the local rotations are characterized by small‐angle modulations rather than large‐scale structural reconstruction. However, a clear difference is observed between the two samples. The low‐BT sample exhibits a sharper and more concentrated distribution, reflecting a more uniform rotation mode and a higher degree of local ordering. In contrast, the high‐BT sample shows a broader and flatter distribution, indicating increased angular dispersion and more pronounced orientation variation among local regions. This demonstrates that BT substitution enhances the randomness and spatial disorder of octahedral rotations. Therefore, the statistical analysis corroborates the trend observed in the real‐space mapping from a probabilistic perspective.

**FIGURE 3:**
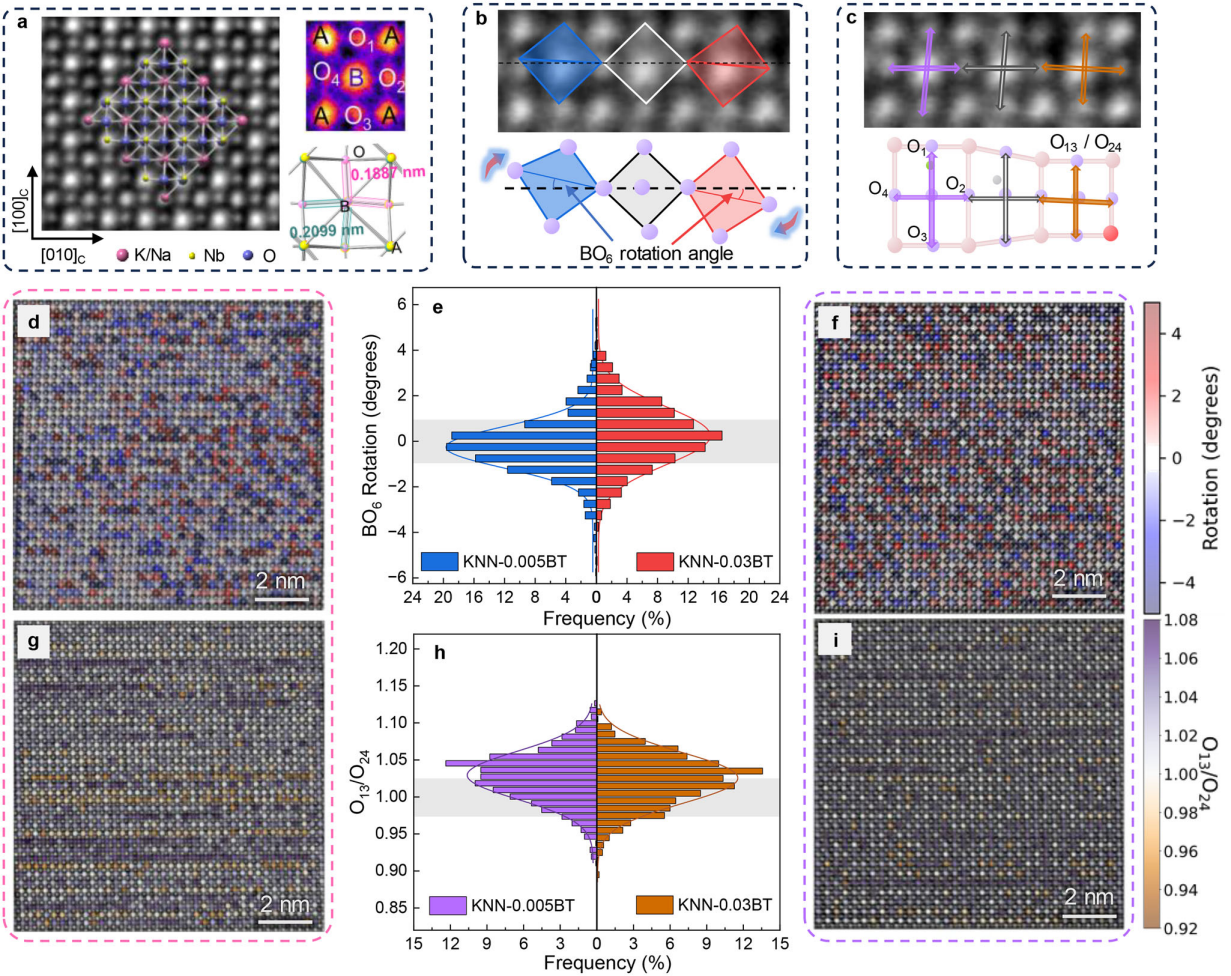
BT‐induced evolution of the oxygen octahedral (BO_6_) rotation framework in KNN ceramics, revealed via iDPC‐STEM image along the [001]_C_ zone axis. (a) Atomic‐resolution iDPC‐STEM image and structural overlay of KNN, highlighting projected oxygen positions and interatomic bond lengths. (b) Schematic illustration of BO_6_ octahedral rotation modes derived from atomic displacement analysis. (c) Schematic definition of local structural descriptors, including octahedral rotation angles and O_13_/O_24_ distortion parameter. (d, f) Spatial distribution maps of BO_6_ rotation angles for KNN‐0.005BT and KNN‐0.03BT, respectively. (e) Comparative statistical distributions of octahedral rotation angles, demonstrating a transition toward enhanced rotation disorder with increasing BT content. (g, i) Spatial maps of the O_13_/O_24_ ratio reflecting local in‐plane octahedral distortion states in KNN‐0.005BT and KNN‐0.03BT. (h) Corresponding statistical distributions of the O_13_/O_24_ parameter, demonstrating enhanced structural disorder upon BT substitution.

In addition to the rotation angles, Figure 3g,i also presents the spatial distribution maps of the O_13_/O_24_ ratio, which is associated with the geometric configuration of the oxygen octahedra, while Figure 3h shows the corresponding statistical distribution. The O_13_/O_24_ ratio is used as a quantitative descriptor of the geometric distortion of oxygen octahedra, where values closer to 1 indicate a more symmetric structure, while larger deviations from 1 correspond to stronger distortion. As shown in Figure 3g–i, increasing BT content leads to a more spatially dispersed and heterogeneous distribution of the O_13_/O_24_ ratio, accompanied by a pronounced broadening of its statistical distribution, indicating that octahedral distortion is concurrently enhanced alongside rotational disorder. This suggests that BT‐induced chemical disorder not only perturbs the rotation of BO_6_ octahedra but also systematically disrupts the local structural coherence of the octahedral framework. In conjunction with the chemical disorder revealed in Figure 2a–c, the rotational disorder of BO_6_ octahedra observed in Figure 3 is fundamentally driven by BT‐induced heterovalent substitutions at both the *A‐* and *B‐*sites. Specifically, the replacement of Nb^5+^ by Ti^4+^ and K^+^/Na^+^ by Ba^2+^ introduces pronounced local compositional fluctuations, charge imbalance, and bond‐length/angle mismatches, thereby disrupting the long‐range lattice ordering of the KNN matrix. Given the corner‐sharing, strongly coupled nature of the BO_6_ framework, such local chemical perturbations progressively break the cooperative rotation, driving a transition from locally ordered to spatially random rotational states.

### 2.3 Synergistic Optimization of Electrostrain and Optical Transparency

With the atomic‐level mechanism of octahedral rotational disorder and local structural evolution established, their fundamental roles in achieving synergistic enhancement of electrostrain performance and optical transmittance are thoroughly elucidated. As shown in Figure 4a,b, all samples exhibit asymmetric strain‐electric field (*S*‐*E*) curves (Figure 4b) and saturated polarization‐electric field (*P*‐*E*) loops under an electric field of 100 kV/cm. Notably, as the BT doping concentration increases, both the maximum polarization (*P*_max_) and strain (*S*_max_) peak at KNN‐0.03BT (Figure 4c), achieving a *P*_max_ of 39.6 µC/cm^2^ and *S*_max_ of 1.2%. This phenomenon stems intrinsically from enhanced polarization vector disorder with rising BT doping, which reduces the switching barrier of defect dipoles (P_D_) [28], facilitates the switching of defect dipoles, and increases the total amount of defect dipoles by enhancing the disorder degree of *A‐*sites [31]. Conversely, overly high doping introduces excessive disorder, leading to a deterioration in the net polarization response. This enhanced disorder also implies a gradual transition to the relaxor state, with the phase transition behavior becoming more diffuse (Figure S8). Figure 4d shows the optical transmittance of polished KNN‐*x*BT ceramics (0.3 mm thickness) in the wavelength range of 200–800 nm. Figure 4e shows considerable optical transmittance at three representative visible‐light wavelengths (420, 620, and 720 nm), where the transmittance reaches its maximum at x = 0.03. Below 400 nm, the transmittance of the material is nearly zero due to the intrinsic crystal absorption caused by interband transitions. Dy A larger bandgap (*E_g_*) demands higher photon energy for electronic transitions, enabling better visible‐light transparency [10]. As shown in Figure 4f, the KNN‐0.03BT composition exhibits the maximum *E_g_* of 2.987 eV. Figure 4g depicts the composition‐dependent evolution of two core properties (asymmetric strain and optical transmittance) in KNN‐*x*BT ceramics. Both properties exhibit synergistic optimization as BT content increases. KNN‐0.03BT achieves the best performance, with an asymmetric strain of 1.2% and visible transmittance enhanced to 60%. To demonstrate the merits of KNN‐0.03BT, the raw comparison data for various transparent ceramic systems are summarized in Table S1 and visualized in Figure 4h, highlighting its concurrent breakthroughs in electromechanical and optical performance. Given that optical transparency also relies on sample thickness, we compare KNN‐0.03BT with representative transparent ceramics at various thicknesses; the detailed data are summarized in Table S2 and visualized in Figure 4i. At the commonly adopted 0.3 mm thickness for polycrystals, the optical transmittance of KNN‐0.03BT approaches that of PMN‐PT ceramic. It overcomes the traditional trade‐off between high strain and high transmittance in polycrystalline ceramics, enabling the development of multifunctional ceramics with both electromechanical response and optical clarity.

**FIGURE 4:**
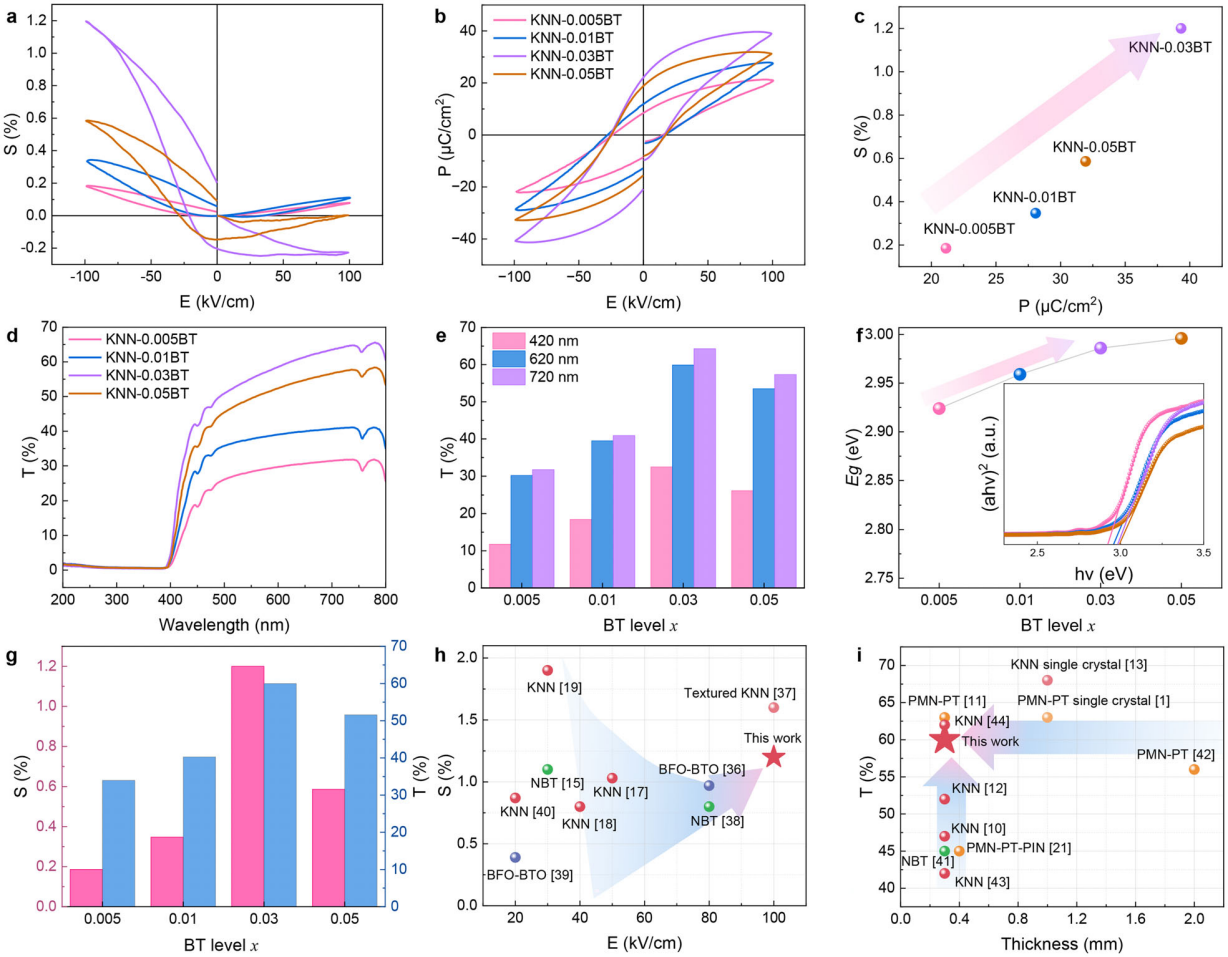
Electrostrain and optical properties of KNN‐*x*BT based ceramics. (a) Room‐temperature strain vs. electric field (*S*‐*E*) loops. (b) Polarization vs. electric field (*P*‐*E*) loops. (c) Strain vs. polarization (*S*‐*P*) curves. (d) Optical transmittance spectra over the light range of 200− 800 nm. (e) Optical transmittance at 420, 620, and 720 nm as a function of the BT concentration *x*. (f) Optical bandgap *E*_g_ as a function of BT content *x*, derived from (αhν)^2^ vs. photon energy (*hν*) Tauc plots (f‐inset). (g) Maximum train and optical transmittance as a function of BT level *x*. (h) Comparison of electrostrain performance (*S*‐*E*) with other reported lead‐free ferroelectric system [15, 17, 18, 19, 36, 37, 38, 39, 40]. (i) Comparison of optical transparency vs. sample thickness with representative single crystals and transparent ceramics [10, 11, 12, 13, 21, 41, 42, 43, 44].

### 2.4 Mechanism for Improved Performance

Building upon the atomic‐scale insights into octahedral rotational disorder, a schematic summary of the underlying structure–property relationship is presented in Figure 5. As illustrated in Figure 5a, BT‐induced chemical disorder drives the BO_6_ octahedral rotation from a locally ordered state to a spatially random distribution, accompanied by lattice distortion and the disruption of long‐range structural coherence [23, 26, 28]. This randomized octahedral framework and the accompanying loss of long‐range coherence serve as structural fingerprints of underlying chemical disorder. Rather than a singular phenomenon, this disorder represents a superposition of two distinct contributions that differ in both their dynamics and functional roles. First, static (compositional) disorder arises from the random occupation of the A‐site sublattice by K^+^, Na^+^ and Ba^2+^ and of the B‐site sublattice by Nb^5+^ and Ti^4+^, which is thermally quenched during sintering [26, 34, 45]. Second, dynamic disorder originates from off‐center positional fluctuations and thermally activated hopping of d° cations (Nb^5+,^ Ti^4+^) within BO_6_ octahedra, driven by the second‐order Jahn‐Teller effect [46]. Static disorder refines the polar domain scale below optical wavelengths to secure optical transparency, whereas dynamic disorder maintains high dipole mobility within a flattened energy landscape to deliver large, low hysteresis electrostrain. Together, these dual contributions lift the conventional trade‐off between optical isotropy and switchable polar anisotropy [47]. This dual disorder redefines the nature of the phase transition itself [1]. Static disorder fragments the displacive framework and sets the nanoscopic length scale of the polar structure [48], while dynamic off‐centering allows dipoles within each fragment to fluctuate among symmetry‐equivalent directions—a characteristic of order‐disorder dynamics that coexists with the displacive soft mode [47]. BT substitution thus couples the displacive transition of KNN to an order–disorder mechanism. The softened TO mode preserves the displacive scaffold, while local random fields and dynamic d^0^ off‐centering generate order‐disorder PNRs that fragment this scaffold at the nanoscale [49]. Consequently, the domain configuration evolves from macro‐sized switchable domains in pure KNN to densely packed, weakly anisotropic PNRs bounded by diffuse domain walls.

**FIGURE 5:**
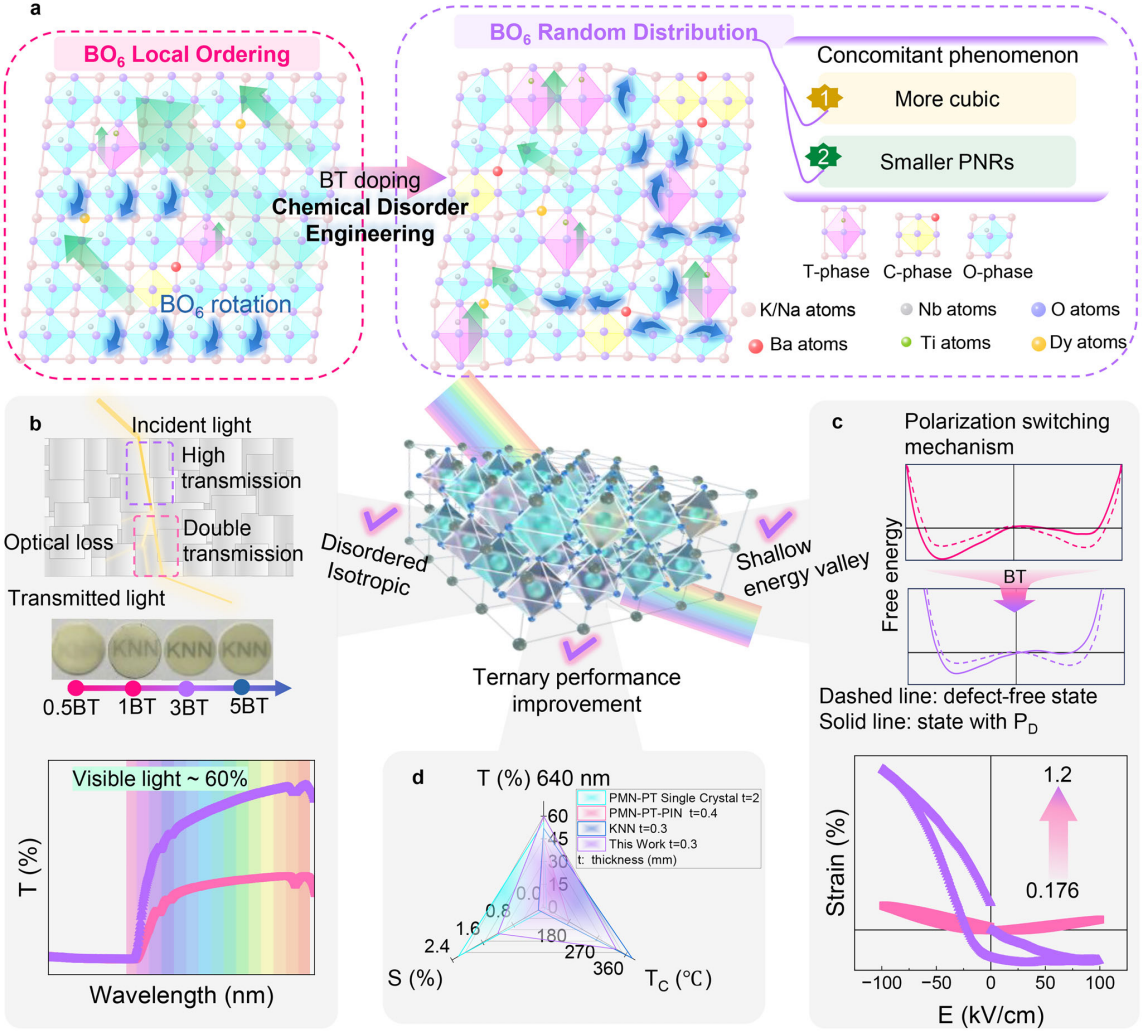
Schematic illustration of chemical‐disorder‐engineered BO_6_ octahedral rotational evolution and its role in the synergistic enhancement of electrostrain and optical transparency in KNN‐*x*BT ceramics. (a) BT‐induced chemical disorder drives the transition of BO_6_ octahedral rotation from local order to spatial randomness, accompanied by the disruption of long‐range polar coherence and domain miniaturization. (b) Optical transmission mechanism: rotational disorder reduces light scattering and optical loss, giving rise to high visible transmittance (∼ 60%). (c) Polarization switching mechanism: chemical disorder flattens the free‐energy landscape to lower the switching barrier, enabling enhanced electrostrain. (d) Ternary performance map comparing with representative materials [12, 21, 42].

The softened displacive mode lowers energy barriers to enhance electrostrain. Meanwhile, order‐disorder nanofragmentation reduces scattering to improve optical clarity. Combining these two structural changes allows the material to achieve both high electrostrain and strong light transmission at the same time [10, 28, 33, 49]. The refined PNRs and reduced long‐range (micrometre‐scale) structural heterogeneity further contribute to improved optical transparency in the KNN‐*x*BT ceramics [11]. As shown in Figure 5c, the free‐energy landscape becomes shallower with reduced thermodynamic barriers, facilitating polarization rotation and switching dynamics [8, 29, 31]. The strategy is thus general rather than system‐specific: any lead‐free ferroelectric with a sufficiently large bandgap, in which disorder can be introduced without destroying the polar order, should be able to exploit the same synergy — as demonstrated here through the well‐tuned BO_6_ octahedral rotation disorder. The performance map shown in Figure 5d, together with the supporting data summarized in Table S3, highlights that the KNN‐0.03BT composition realizes a favorable triangular balance among large electrostrain, high optical transparency, and decent T_C_. This performance balance does not violate the intrinsic physical constraints of materials, rather, it originates from the synergy between displacive softening and order–disorder nanofragmentation. This strategy breaks the conventional technical bottleneck of traditional polycrystalline ferroelectric ceramics. Overall, this work establishes a clear mechanistic framework linking chemical disorder, octahedral rotational evolution, and multifunctional property enhancement, providing a viable strategy for the design of advanced transparent ferroelectric materials. The material cost associated with niobium is largely mitigated by the high value density of the targeted transparent electro‐opto‐mechanical devices, where the simultaneous realization of high optical transparency and electrostrain offers significant integration advantages over single‐function alternatives [32].

## 3 Conclusions

In conclusion, this work realizes effective structural modulation and performance optimization of lead‐free KNN‐xBT ceramics, systematically clarifying the intrinsic correlation between octahedral rotation framework and macroscopic functional properties. Chemical disorder induced by BT doping achieves synergistic enhancement of electrostrain and optical transmittance by regulating the BO_6_ octahedra rotation mode, which triggers polarization disorder and generates refined PNRs. The KNN‐0.03BT ceramic simultaneously delivers a large asymmetric strain of 1.2% under a high electric field and a high visible‐light optical transmittance up to 60%. Such competitive performance is comparable to representative high‐performance lead‐based single crystals and textured ceramics, breaking the long‐standing bottleneck of lead‐free ceramics where large electrostrain and high optical transmittance are mutually exclusive and hard to achieve simultaneously.

## 4 Methods

### 4.1 Sample Preparation

(1‐*x*)(K_0.5_Na_0.5_)_0.98_Dy_0.02_NbO_3_‐*x*BaTiO_3_ (*x* = 0.005, 0.01, 0.03, 0.05) ceramics were synthesized via the traditional high temperature solid state method. First, the raw materials of K_2_CO_3_ (Aladdin, 99.99%), Na_2_CO_3_ (Aladdin, 99.99%), Nb_2_O_5_ (Macklin, 99.99%), Dy_2_O_3_ (99.99%), BaCO_3_ (Aladdin, 99.99%), and TiO_2_ (Aladdin, 99.99%) were weighed according to the stoichiometry before grinding with zirconia balls in ethanol for 24 h. After being dried and calcined at 850°C, the powders were milled again for 12 h and dried at 60°C. Finally, pellets measuring 12 mm in diameter with 8 wt.% PVA were prepared by pressing the pre‐calcined powders. After removing the PVA binder at 550°C for 6 h, the obtained pellets were sintered in a muffle furnace at 1150–1170°C for 4 h.

### 4.2 Characterizations

The phase purity and structure were examined by powder x‐ray diffraction (XRD) (Rigaku SmartLab SE, Cu‐Kα radiation) in the 9M range from 10° to 90°. In order to detect the low‐level impurity phase, an integral linear detector was employed with scanning step of 2° during the XRD measurements. The Rietveld refinement method was used to analyze the purity and cell parameters by refining the measured XRD patterns. Raman spectra were recorded with a Renishaw spectrometer, using a 532 nm diode laser, and characterization was performed in the range of 100–1000 cm^−1^. The microstructure features of the samples were observed using a scanning electron microscope (SEM, Zeiss Gemini 500). Optical absorption and transmittance were analyzed by UV–vis diffuse reflectance spectroscopy (Metash UV‐9000S, BaSO_4_ as reference). X‐ray photoelectron spectroscopy (XPS, Thermo) was utilized to gather comprehensive valence state information, revealing the oxidation states and chemical environments of the constituent elements. For electrical properties measurement, the ceramics were polished to 0.3 mm thickness and sputtered with a 1 mm diameter Au electrode. Gold electrodes were sputtered on both sides of the samples for the measurement of polarization (*P*) and Strain (*S*) vs. electric field (*E)* using a ferroelectric test system (aixACCT TF3000). The dielectric performance of the samples, including capacitance, dielectric loss, and conductivity, was investigated using an impedance analyzer (Agilent 4990A‐120).

Atomic‐resolution imaging and analysis were performed using a spherical‐aberration‐corrected transmission electron microscope (Thermo Fisher Scientific Spectra 300) operated at 300 kV. High‐angle annular dark‐field STEM (HAADF‐STEM) was conducted with a probe current of 70 pA, a beam convergence semi‐angle of 17.8 mrad, and detector collection angles of 51–200 mrad. To optimize spatial resolution and reduce beam‐induced artifacts, images were acquired using a 2048 pixel × 2048 pixel matrix with 5 µs·pixel^−1^ dwell time, combined with dynamic beam scanning compensation to counteract sample drift. For light‐element‐sensitive structural characterization, integrated differential phase contrast STEM (iDPC‐STEM) was employed under a reduced probe current (5 pA) while retaining the 17.8 mrad convergence semi‐angle. The phase‐sensitive detector was configured with restricted collection angles (5–25 mrad) to enhance contrast for low‐atomic‐number elements. High‐fidelity imaging utilized a 2048 pixel × 2048 pixel raster scan and 2 µs·pixel^−1^ dwell time, ensuring minimal radiation damage while resolving sub‐ångström structural features.

### 4.3 Statistical Analysis

No artificial data transformation, normalization, or outlier screening was performed. All fitting and structural analyses were implemented based on raw experimental data without manual modification. All quantitative statistical data in bar graphs are presented as mean ± standard deviation (mean ± SD). No data smoothing or nonlinear optimization was conducted. Each group of material samples was tested with no fewer than three independent replicates. For STEM lattice analysis, more than 500 B‐site atomic columns were statistically fitted per group to ensure analytical reliability. Targeted fitting methods were adopted for different characterization data. Raman spectra were deconvoluted using a Lorentzian‐Gaussian hybrid function. XPS peak fitting was performed with a pure Gaussian function. XRD patterns were refined via full‐profile refinement using GSAS‐II software. STEM atomic localization was realized by a self‐developed Python‐based Multiple‐Ellipse Fitting (MEF) algorithm. Specifically, a local ROI was cropped for each atomic spot, eight equally spaced intensity thresholds were set to extract closed intensity contours, and constrained least‐squares ellipse fitting was applied to each contour to obtain sub‐pixel atomic positions. All bar quantitative data were fitted via the GCAS method. No significance test or *p*‐value analysis was required, as all results were obtained from deterministic structural fitting.

## Author Contributions

W. Hu conceived and designed the research. X. Luo synthesized the samples and performed the structure, property, and electron microscopy characterizations. X. Luo and J. Xie conducted the STEM measurement and structural analysis. X. Luo, J. Wang, and Y. Tan conducted the data analysis and wrote the manuscript with input from all authors. W. Hu reviewed, polished, and revised the manuscript.

## Conflicts of Interest

The authors declare no conflicts of interest.

## Supporting information




**Supporting File**: advs77952‐sup‐0001‐SuppMat.docx.

## Data Availability

The data that support the findings of this study are available from the corresponding author upon reasonable request.
