# Supplementary material for: Large Electrostrain and High Optical Transmittance via Oxygen Octahedron Rotation Disordering in KNN Ceramics
Source: Adv Sci (Weinh). 2026 Oct 11:e77952. Online ahead of print. doi: 10.1002/advs.77952 (PMC13654865; doi:10.1002/advs.77952)
Supplement: Supplementary file 1 [file ADVS-9999-e77952-s001.docx]

**Supplementary Information**

**Large Electrostrain and High Optical Transmittance via Oxygen Octahedron Rotation Disordering in KNN Ceramics**

Xia Luo,^1#^ Jian Wang,^1#^ Yu Tan,^1,3^ Jiyang Xie^1^, Thamil Selvi Velayutham^2,4^ and Wanbiao Hu^1,2,3*^

^1^ Yunnan Key Laboratory of Electromagnetic Materials and Devices, National Center for International Research on Photoelectric and Energy Materials, School of Materials and Energy, Yunnan University, Kunming 650091, P. R. China

^2^ School of Engineering, Yunnan University, Kunming 650091, P. R. China

^3^ Southwest United Graduate School, Kunming 650092, P. R. China

^4^ Low Dimensional Materials Research Centre (LDMRC), Department of Physics, Faculty of Science, Universiti Malaya, 50603 Kuala Lumpur, Malaysia

E-mail: [huwanbiao@ynu.edu.cn](mailto:huwanbiao@ynu.edu.cn)

^#^ These authors contributed equally to this work.

**Fig. S1. Crystal structure evolution of KNN-*x*BT ceramics.** (a) XRD Rietveld refinement. (b) In-situ temperature-dependent XRD patterns for KNN-0.03BT. Clearly, all samples present nearly identical XRD diffraction features, including consistent 2θ angles and relative diffraction intensities. High-resolution fine scanning further confirms the absence of secondary impurity phases throughout the tested range. It sufficiently indicates that the (1-*x*)(K_0.5_Na_0.5_)_0.98_Dy_0.02_NbO_3_-*x*BaTiO_3_ (*x*=0.005, 0.01 0.03, 0.05) system does not alter the bulk crystal structure of KNN ceramic. The crystal structure belongs to a typical distorted perovskite configuration, which is composed of mixed perovskite phases with coexisting *Amm2* and *P4mm* space groups. The structure for KNN-0.03BT is thermally stable in the temperature range of 20 ^o^C ~450 ^o^C (Fig. S1b).

**Fig. S2. Raman spectra of KNN-*x*BT ceramics.** The observed modes can be separated into external translational modes which relate to A-site cation located mainly at the frequency less than 200 cm^-1^ and the internal vibrational modes of BO_6_ octahedra located at the frequency range of 200-1000 cm^-1^, respectively. The BO_6_ consists of six normal vibration Γ=1A_1g_(*v_1_*) + 1E_g_(*v_2_*) + 2F_1u_(*v_3_*, *v_4_*) + F_2g_(*v_5_*) + F_2u_(*v_6_*), where 1A_1g_ (*ν_1_*)+1E_g_ (*ν_2_*)+2F_1u_ (*ν_3_*) represents stretching modes while the others are bending modes. The ν_1_+ν_5_ mode at about 860 cm^-1^ is attributed to the anharmonic coupling of ν_1_ and ν_5_ modes. These Raman modes reveal light variations in wavenumber position, relative intensity ratio and peak width with various BT doping contents. These deviations are too weak to reflect a macroscopic phase transition, and mainly stem from local atomic‑scale structural disorder, which indicates that the long‑range average lattice structure and chemical‑bond vibrations of KNN ceramics are well maintained.

**Fig. S3. Statistical analysis of morphology and grain size of KNN-*x*BT ceramics.** SEM (Scanning Electron Microscopy) micrographs, grain size distribution histograms, and average grain size evolution of KNN-*x*BT ceramics with different BT contents (*x* = 0.005, 0.01, 0.03, 0.05). All samples exhibit dense and homogeneous microstructures with unimodal grain size distributions. The average grain size remains stable at approximately 180-210 nm and shows no significant variation with increasing BT content, indicating that BT doping has no pronounced effect on grain growth. The narrow grain size distribution and average size of 180-210 nm suggest that multiple spontaneous polarization orientations can coexist within each grain. The 2at.% Dy doping serves as a sintering aid to promote ceramic densification. In addition, Dy^3+^ preferentially occupies the A‑site (K^+^/Na^+^) of the perovskite lattice. Ionic‑radius mismatch induces local lattice distortion, and Dy segregation at grain boundaries suppresses grain‑boundary migration and abnormal grain growth, resulting in refined and uniform grains for all four investigated compositions.

**Fig. S4. Atomic-scale elemental distribution for KNN-0.03BT.** (a)-(h) HAADF and EDS mapping. (i) Atomic column intensity profiles extracted from HAADF imaging, providing quantitative insights into atomic arrangement. The results demonstrate that all elements are homogeneously distributed in the lattice without obvious segregation or phase separation, confirming the successful incorporation of dopants into the perovskite lattice.

**Fig. S5. Spatial distribution and intensity statistics of displacement polarization vector field.** Figs. S5a,c show the HAADF-STEM images acquired along the [001]_C_ zone axis. The pink boxed region in Fig. S5c corresponds to the marked area in Fig. 2a in the main text where a high-resolution atomic-scale STEM-HAADF image is given. Figs. S5b,d display the lattice displacement vector maps corresponding to Figs. S5a,c, respectively. Statistical analysis of cation displacements and phase ratio bar chart for graphs Figs. S5e,f. Compared with KNN-0.005BT, the KNN-0.03BT ceramic exhibits more diverse polarization displacement orientations and larger displacement magnitudes, which is beneficial to polarization switching and strain response under an applied electric field.

**Fig. S6. (a) HAADF-STEM image along the [110]_C_ direction and (b) Statistical analysis of displacements for KNN-0.03BT.** The results confirm that the polarization displacement magnitudes of KNN-0.03BT along the [110]_C_ direction are consistent with those obtained from the [100]_C_ direction, verifying the statistical reliability of the atomic displacement distribution.

**Fig. S7. iDPC-STEM images of KNN-0.005BT and KNN-0.03BT.** (a) and (b) are the crystal morphology images of the collected regions for KNN-0.005BT and KNN-0.03BT. (c) and (d) are the iDPC-STEM image for KNN-0.005BT and KNN-0.03BT. Both samples exhibit high-quality, high-resolution iDPC images of the [001]_C_ lattice, providing reliable data support for subsequent atomic-scale phase analysis and fitting.

**Fig. S8. Dielectric properties.** (a) Temperature‑dependent dielectric and loss for KNN‑*x*BT. (b) Relationship between ln(1/ε - 1/ε_m_) and ln(T - T_m_) at 1kHz for the KNN-*x*BT. With increasing BT content, the Curie temperature (T_C_) decreases from 356 °C (KNN-0.005BT) to 330 °C (KNN-0.05BT). The relationship is as follows[1]:

$$\frac{1}{\varepsilon_{r}}-\frac{1}{\varepsilon_{m}}=\frac{{(T-T_{m})}^{\gamma}}{C}$$

where ε_r_ is the relative permittivity at temperature T, ε_m_ is the maximum value of the relative permittivity (i.e. the value at the dielectric peak), T is the absolute temperature, T_m_ is the temperature at which ε_r_ reaches its maximum, C is the Curie–Weiss constant, and γ (1 ≤ γ ≤ 2) is the diffuseness exponent characterizing the degree of dielectric relaxation: γ = 1 corresponds to a normal ferroelectric obeying the classical Curie–Weiss law, whereas γ = 2 corresponds to an ideal relaxor with a completely diffuse phase transition. Meanwhile, the broadening of dielectric peaks and enhanced frequency dispersion indicate strengthened relaxor behavior, with the fitted Curie-Weiss relaxation factor γ increasing from 1.49 to 1.92. These results confirm that the enhanced relaxor characteristics originate from increased microstructural disorder induced by multiphase coexistence.

**Table S1. Asymmetric strain (S) comparison of Pb-free ferroelectric materials**

| Composition | Crystal morphology | E (kV/cm) | S (%) | Ref. |
| --- | --- | --- | --- | --- |
| BFO-BTO | Polycrystals | 80 | 0.97 | [2] |
| BFO-BTO | Polycrystals | 20 | 0.39 | [3] |
| NBT | Polycrystals | 80 | 0.8 | [4] |
| NBT | Polycrystals | 30 | 1.1 | [5] |
| KNN | Polycrystals | 50 | 1.03 | [6] |
| KNN | Polycrystals | 30 | 1.9 | [7] |
| KNN | Polycrystals | 20 | 0.87 | [8] |
| KNN | Texture | 100 | 1.6 | [9] |
| KNN | Polycrystals | 100 | 1.2 | This work |

**Table S2. Optical transmittance (visible light) of ferroelectric materials**

| Composition | Crystal morphology | Thickness (mm) | T (%)^*^ | Ref. |
| --- | --- | --- | --- | --- |
| PMN-PT | Single crystal | 1 | 63 | [10] |
| PMN-PT-PIN | Polycrystals | 0.3 | 45 | [11] |
| PMN-PT | Polycrystals | 2 | 56 | [12] |
| PMN-PT | Polycrystals | 0.3 | 63 | [13] |
| NBT | Polycrystals | 0.3 | 45 | [1] |
| KNN | Polycrystals | 0.3 | 47 | [14] |
| KNN | Polycrystals | 0.3 | 42 | [15] |
| KNN | Polycrystals | 0.3 | 62 | [16] |
| KNN | Single crystal | 1 | 68 | [17] |
| KNN | Polycrystals | 0.3 | 60 | This work |

**Table S3. Transmittance and Curie temperature (T_C_) of ferroelectric materials**

| Composition | Crystal morphology | Thickness (mm) | T (%) | T_C_ (℃) | Ref. |
| --- | --- | --- | --- | --- | --- |
| PMN-PT | Single crystal | 2 | 56 | 109 | [12] |
| PMN-PT-PIN | Polycrystals | 0.3 | 45 | 180 | [11] |
| KNN | Polycrystals | 0.3 | 52 | 380 | [18] |
| KNN | Polycrystals | 100 | 1.2 | 330 | This work |

Tables S1-S3 compare the key performance metrics of this work with literature-reported lead-free ferroelectric ceramics. The results show that our KNN-based polycrystalline ceramic achieves an asymmetric electrostrain of 1.2% at 100 kV/cm, comparable to textured KNN ceramics. Meanwhile, it exhibits 60% visible-light transmittance at thickness of 0.3 mm and a high Curie temperature of 330 ℃, demonstrating a good balance between strain performance, optical transparency, and thermal stability.

**References**

[1]	X. Zeng, X. Jiang, J. Lin, Q. Lin, S. Wang, Y. Wu, X. Wu, M. Gao, C. Zhao, T. Lin, L. Luo, C. Lin, *Chemical Engineering Journal* **2024**, *496*, <https://doi.org/10.1016/j.cej.2024.154150>.

[2]	W. Li, C. Zhou, J. Wang, C. Yuan, J. Xu, Q. Li, G. Chen, J. Zhao, G. Rao, *Materials Today Chemistry* **2022**, *26*, 101237, <https://doi.org/10.1016/j.mtchem.2022.101237>.

[3]	J. Lin, X. Guo, B. Liu, Z. Qian, J. Chen, T. Sun, J. Liao, J. Li, Z. Hong, Y. Wu, X. Yu, Y. Huang, *Advanced Functional Materials* **2025**, *36* (18), e22553, <https://doi.org/10.1002/adfm.202522553>.

[4]	X. Nie, R. Jing, F. Chen, L. Zhang, Y. Yang, Z. Yang, S. Zhang, H. Jiao, H. Zhang, H. Yang, L. Jin, *Advanced Functional Materials* **2025**, *36* (11), e13360, <https://doi.org/10.1002/adfm.202513360>.

[5]	G. Huangfu, J. Wang, H. Zhang, J. Chen, Z. Liu, Y. Guo, *Nano Letters* **2024**, *24* (39), 12148, <https://doi.org/10.1021/acs.nanolett.4c03042>.

[6]	X. Zhou, J. Zhang, H. Luo, Y. Zhang, S. Tang, H. Huang, X. Yuan, M. Song, H. Qi, D. Zhang, *Nature Communications* **2024**, *15* (1), 6625, <https://doi.org/10.1038/s41467-024-51082-6>.

[7]	Z. Xu, X. Shi, Y.-X. Liu, D. Wang, H.-C. Thong, Y. Jiang, Z. Sha, Z. Li, F.-Z. Yao, X.-X. Cai, H.-F. Huang, Z. Xu, X. Jin, C.-B.-W. Li, X. Zhang, X. Ren, Z. Dong, C. Wu, P. Kabakov, F. Zhu, F. Chen, P. Tan, H. Tian, H. Sha, R. Yu, B. Xu, W. Gong, X. Wang, J.-F. Li, S. J. Skinner, M. Li, H. Huang, S. Zhang, K. Wang, *Nature Materials* **2025**, *24* (4), 565, <https://doi.org/10.1038/s41563-024-02092-8>.

[8]	J. Wang, B. Wang, H. Zhang, S. Zhang, Y. Guo, *Advanced Materials* **2024**, *36* (33), 2404682, <https://doi.org/10.1002/adma.202404682>.

[9]	L. Lai, B. Li, S. Tian, Z. Zhao, S. Zhang, Y. Dai, *Advanced Materials* **2023**, *35* (29), 2300519, <https://doi.org/10.1002/adma.202300519>.

[10]	C. Qiu, B. Wang, N. Zhang, S. Zhang, J. Liu, D. Walker, Y. Wang, H. Tian, T. R. Shrout, Z. Xu, L.-Q. Chen, F. Li, *Nature* **2020**, *577* (7790), 350, <https://doi.org/10.1038/s41586-019-1891-y>.

[11]	F. Zheng, X. Tian, Z. Fang, J. Lin, Y. Lu, W. Gao, R. Xin, D. Fu, Y. Qi, Z. Ma, W. Ye, Y. Qin, X. Wang, Y. Zhang, *ACS Applied Materials & Interfaces* **2023**, *15* (5), 7053, <https://doi.org/10.1021/acsami.2c19865>.

[12]	J. Song, C. He, W. Qiu, Z. Miao, Y. Shi, C. Deng, L. Liu, M. Xia, Q. Li, Y. Lu, G. Zhang, *Journal of the American Ceramic Society* **2026**, *109* (3), e70596, <https://doi.org/10.1111/jace.70596>.

[13]	M. Yuan, D. Fu, Y. Wang, C. Li, G. Zhu, D. Xu, W. Shao, Y. Li, W. Ye, Y. Zhang, *Ceramics International* **2026**, *52* (9), 12726, <https://doi.org/10.1016/j.ceramint.2026.01.415>.

[14]	Q. Lin, X. Zeng, X. Huang, J. Lin, M. Gao, C. Zhao, C. Lin, X. Wu, *Chemical Engineering Journal* **2025**, *520*, 165886, <https://doi.org/10.1016/j.cej.2025.165886>.

[15]	Z. Gong, H. Yue, K. Fang, K. Guo, K. Li, C. Guo, H. Zhang, Z. Deng, Z. Liu, B. Xie, P. Mao, J. Lu, S. Guo, K. Yao, F. E. H. Tay, *Journal of Materiomics* **2025**, *11* (4), 100993, <https://doi.org/10.1016/j.jmat.2024.100993>.

[16]	Q. Lin, J. Shen, X. Zeng, X. Huang, W. Shi, B. Sa, H. Yan, M. Gao, C. Zhao, C. Lin, X. Wu, J. Lin, J. Zhai, *ACS Nano* **2026**, *20* (24), 17803, <https://doi.org/10.1021/acsnano.6c08516>.

[17]	F. Yin, L. Liu, M. Zhu, J. Lv, X. Guan, J. Zhang, N. Lin, X. Fu, Z. Jia, X. Tao, *Advanced Optical Materials* **2022**, *10* (23), 2201721, <https://doi.org/10.1002/adom.202201721>.

[18]	J. Lin, Y. Wang, R. Xiong, B. Sa, C. Shi, J. Zhai, Z. Fang, K. Zhu, F. Yan, H. Tian, G. Ge, G. Li, H. Bai, P. Wang, Y. Zhang, X. Wu, *Acta Materialia* **2022**, *235*, 118061, <https://doi.org/10.1016/j.actamat.2022.118061>.
